# A Delphi consensus to identify the key screening tests/questions for a digital neurological examination for epidemiological research

**DOI:** 10.1007/s00415-024-12254-y

**Published:** 2024-02-20

**Authors:** Vasco Ribeiro Ferreira, Carol Brayne, Paolo Ragonese, Carlos Ketzoian, Marta Piccioli, Lorenzo Tinti, Carlo Casali, Cherubino di Lorenzo, Claudia Ramos, João Azevedo, Adriana Gomes, Roderick Stewart, Hein Haas, Stan Hoppenbrouwer, Esther Metting, Valentina Gallo

**Affiliations:** 1 Department of Sustainable Health, University of Groningen, Campus Fryslân, Wirdumerdijk 34, 8911 CE Leeuwarden, The Netherlands; 2 Cambridge Public Health, University of Cambridge, Cambridge, United Kingdom; 3 Department of Biomedicine, Neuroscience and Advanced Diagnostics (BIND), University of Palermo, Palermo, Italy; 4 Institute of Neurology, School of Medicine, Neuroepidemiology Section, University of the Republic, Montevideo, Uruguay; 5 UOC of Neurology, PO San Filippo Neri, ASL Roma 1, Rome, Italy; 6 Laboratory of Neurology, Mario Negri Institute for Pharmacological Research (IRCCS), Milan, Italy; 7 Department of Medico-Surgical Sciences and Biotechnologies (SBMC), University Rome Sapienza, Rome, Italy; 8 Grupo de Neurociencias de Antioquia (GNA), Faculty of Medicine, University of Antioquia, Medellín, Colombia; 9 Grupo de Neuropsicología y Conducta (GRUNECO), Faculty of Medicine, University of Antioquia, Medellín, Colombia; 10 Agrupamento de Centros de Saúde de Gaia, Unidade de Saúde Familiar Nova Salus, Vila Nova de Gaia, Portugal; 11 Hospital de Braga, EPE, Braga, Portugal; 12 Parkinson’s UK, London, United Kingdom; 13 Parkinson Vereniging, Bunnik, The Netherlands; 14 University Medical Center Groningen, Groningen, The Netherlands; 15 Faculty of Economics and Business, University of Groningen, Groningen, The Netherlands

**Keywords:** Epidemiology, eHealth, Neurological diseases, Neuroepidemiology

## Abstract

**Background:**

Most neurological diseases have no curative treatment; therefore, focusing on prevention is key. Continuous research to uncover the protective and risk factors associated with different neurological diseases is crucial to successfully inform prevention strategies. eHealth has been showing promising advantages in healthcare and public health and may therefore be relevant to facilitate epidemiological studies.

**Objective:**

In this study, we performed a Delphi consensus exercise to identify the key screening tests to inform the development of a digital neurological examination tool for epidemiological research.

**Methods:**

Twelve panellists (six experts in neurological examination, five experts in data collection—two were also experts in the neurological examination, and three experts in participant experience) of different nationalities joined the Delphi exercise. Experts in the neurological examination provided a selection of items that allow ruling out neurological impairment and can be performed by trained health workers. The items were then rated by them and other experts in terms of their feasibility and acceptability.

**Results:**

Ten tests and seven anamnestic questions were included in the final set of screening items for the digital neurological examination. Three tests and five anamnestic questions were excluded from the final selection due to their low ratings on feasibility.

**Conclusion:**

This work identifies the key feasible and acceptable screening tests and anamnestic questions to build an electronic tool for performing the neurological examination, in the absence of a neurologist.

## Background

The burden of neurological diseases is an increasing public health concern. In the past 30 years, neurological diseases have been consistently ranked as the leading cause of disability-adjusted life-years (DALYs), and among the leading causes of death worldwide, with a burden expected to further steadily increase in the next decades [1]. The leading contributors of the increase of DALYs attributed to neurological diseases are stroke, headache disorders, and dementia, with the biggest impact in low- to middle-income countries, where nearly 80% of neurological deaths occur to date [2]. Neurological diseases have also been listed as one of the highest contributors to health expenditure, nearing 270 billion euros in Europe alone [3].

For most neurological diseases, there are no curative treatments yet, and thus preventive strategies are key to reduce their burden [4]. The most recent World Health Organization (WHO) global action plan focuses on prevention and control of neurological diseases: action points cover governmental and local levels, and particular emphasis is put on investing, promoting and disseminating research on neurological diseases [5].

To date, the risk factor profile for many neurological diseases remains elusive, preventing effective public health campaigns. However, continuous research on large populations was revealed to be key in better understanding risk factor profiles, ultimately transforming the research landscape on neurological diseases [6, 7], as seen for example in the case of Alzheimer’s disease [8, 9]. Identification of key risk factors, in fact, not only poses the basis for preventive campaigns, but also gives important clues on potential aetiological neuropathological mechanisms whose identification is key for the discovery of diagnostic and treatment targets [9] using for example biomarkers [10]. Therefore, population-based epidemiological studies investigating risk factors for neurological diseases are urgently needed. Current experiences come mainly from the Global North, i.e. the USA [11–15], Europe [6, 7, 16–18], and China [19, 20]. Widening the source population for such studies by extending them to different geographical locations would allow drawing a complete picture of the profile risk by including a wider range of exposure levels.

However, researching neurological diseases in population-based epidemiological studies is limited by case ascertainment. Diagnosis of many neurological diseases relies on costly resources, such as sophisticated medical equipment and/or an expert neurologist to perform a neurological examination. Despite available access to routine records, even in extensively studied populations such as those in the Global North, ascertainment of neurological outcomes can be challenging, as it relies on the judgement of trained neurologists and multidisciplinary consensus approaches for validation of diagnoses [21, 22]. Overall, these limitations become even more restrictive in hard to reach regions and populations, preventing epidemiological studies of neurological outcomes [23], and leading to inequities in access to research and inability to represent communities’ needs accurately. The rapid emergence and development of electronic health (eHealth) has been welcomed by many as a potential alternative to improve this situation in a sustainable and accessible way [24].

In the past decade, several eHealth tools have been devised to collect neurological data (e.g. screening and monitoring) for research (for a comprehensive review of software tools, refer to [25]). However, these tools are limited to data collection of either one function or one disorder. In addition, most fail to provide an adequate means of accessibility (e.g. a proper description, a uniform resource locator—URL, etc.). These tools demonstrate that it is feasible to devise digital tools for assessing the neurological function, but their narrow focus limits their use as basis for case ascertainment in population-based studies. Some scales devised to monitor specific neurological disease progression (i.e. the NIH Stroke Scale [26] and the Glasgow Coma Scale [27]) are currently used in clinical practice by trained personnel. There are no data on their validity in detecting neurological impairment at the population level.

As part of the background work for devising a new eHealth research tool to assess the neurological function in epidemiological studies, a selection of key tests and questions to guide data collection is needed. To devise an assessment that is as parsimonious as possible, the key screening questions that can rule out the largest number of neurological signs and symptoms combined need to be identified. In addition, to what extent they can be uniquely and correctly interpreted by a health assessor who is not a clinical neurologist and to what extent they are acceptable to participants are key features which need to be accounted for. A Delphi consensus exercise was conducted to identify the key items of the neurological examination and their feasibility and acceptability when administered to the general population in a context of an epidemiological study. In this paper, the purpose, methods and results of this Delphi consensus are reported.

## Methods

### Context

This Delphi consensus is part of a wider study aimed at developing an eHealth tool to assess neurological impairment at the population level, in the absence of a neurologist, to be used for epidemiological research. As a first step, a systematic review mapped existing eHealth software tools assessing one or more neurological functions [25]. This Delphi exercise represents a step further of the theoretical work needed for the development of the final eHealth tool—the NeuroEpiTool.

This study aimed to reach a consensus among experts on what the screening tests/anamnestic questions are to assess neurological function to maximise the observer’s ability to rule out the largest number of neurological signs and symptoms. Following a parsimonious principle, when all items are negative, neurological impairment can be ruled out in a participant; and if positive, further tests need to be administered. The NeuroEpiTool will then undergo firstly a comparison study at the individual level against a clinical neurological examination, followed by a validation phase at the population level to compare the ability of specific combinations of signs and symptoms to predict a neurological disease.

**Fig. 1:**
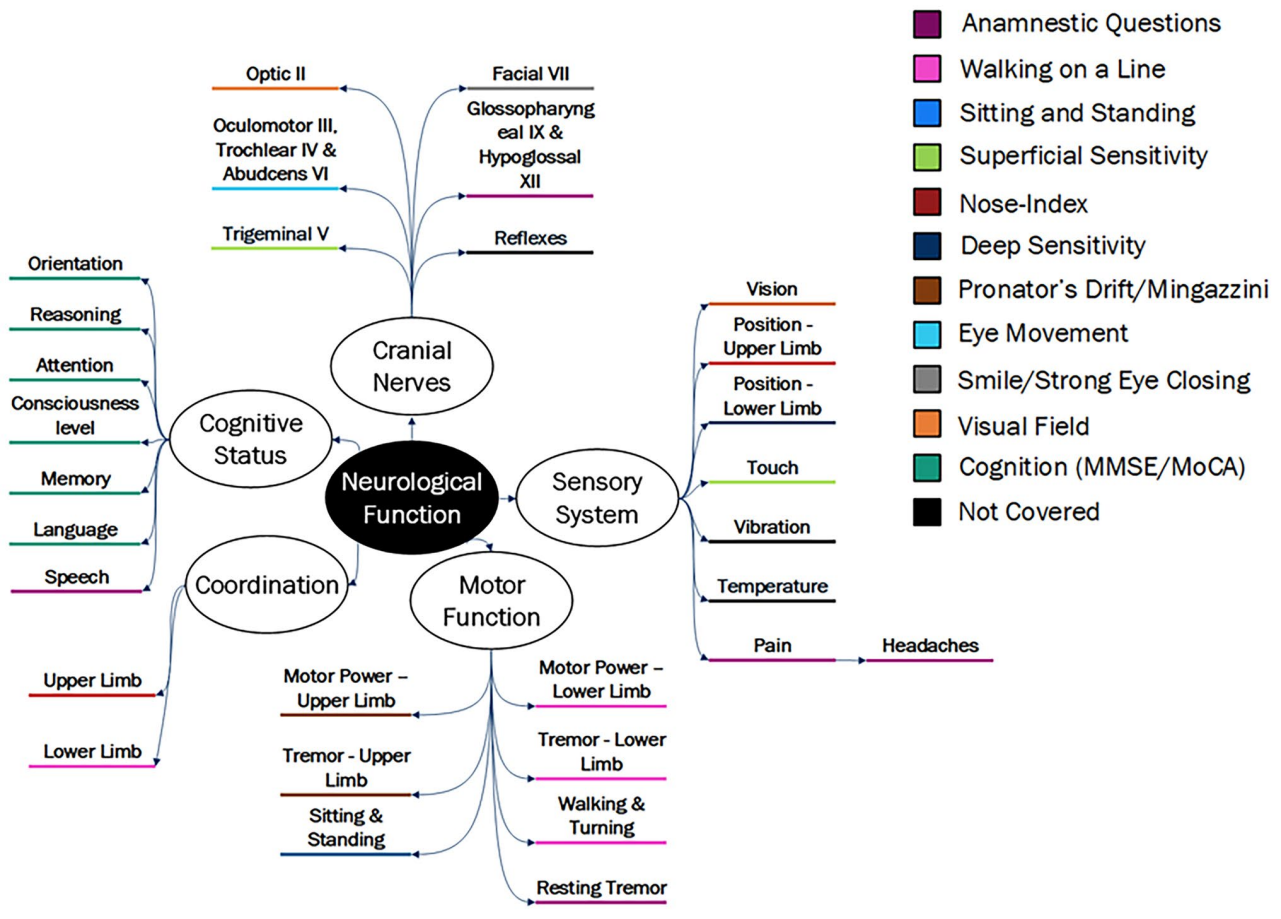
Mapping of the neurological function covered by the selected consensus items

Given the general context in which this research develops and the need to interpret the data coming from the NeuroEpiTool without the presence of a neurologist or any instrumental diagnostic procedure, we deliberately chose to approach the neurological examination by function, rather than anatomical location. A map of the neurological function was compiled and provided to the expert for guidance (see Fig. 1). The final selection of items identified needed to meet both the feasibility and acceptability criteria, i.e. they had to be feasible to perform and interpret by a trained health worker and acceptable by potential research participants.

### Defining the neurological examination

The map of neurological function was organised according to five core domains: cognitive status, cranial nerve function, motor strength, sensory system and coordination [28]. Each domain branches into several functions or subfunctions (e.g. orientation and attention in cognitive status, upper and lower limb motor function) which can be selectively impaired. As the map was based on function alone, some conditions or diseases may be left out of the mapping (e.g. epilepsy); however, experts were invited to provide complementary tests/questions that screen conditions not represented in the map.

### Sample characteristics

Given the overarching scope of the Delphi exercise, we identified three different groups of experts: in the neurological examination, in data collection, and in participant experience. The original aim was to gather a sample of approximately 15 experts. Experts were invited through personal network, identification from the scientific literature, and snowballing. Special attention was posed in widening the geographical location and to ensure gender balance of the participants. Experts were invited to self-identify as expert in one or more roles.

Experts in the neurological examination (i.e. clinical neurologists) were asked to identify the screening tests/anamnestic questions for assessing neurological function. A total of six experts were involved, five from Italy and one from Uruguay (CC, CDL, CK, LT, MP, PR).

Experts in data collection (i.e. researchers with experience of data collection in the field) were asked to assess the feasibility of performing and interpreting each test identified by the experts in the neurological examination. A total of five were involved, two from Portugal, one from Italy, one from Colombia, and one from Uruguay (AG, CR, CK, JA, LT). Two experts in data collection were also experts in the neurological examination.

Experts in participant experience (i.e. people living with a neurological disorder) were asked to assess the acceptability of each test. A total of three were involved, two from the Netherlands and one from the UK (HH, RS, SH).

### Delphi rounds

**Fig. 2:**
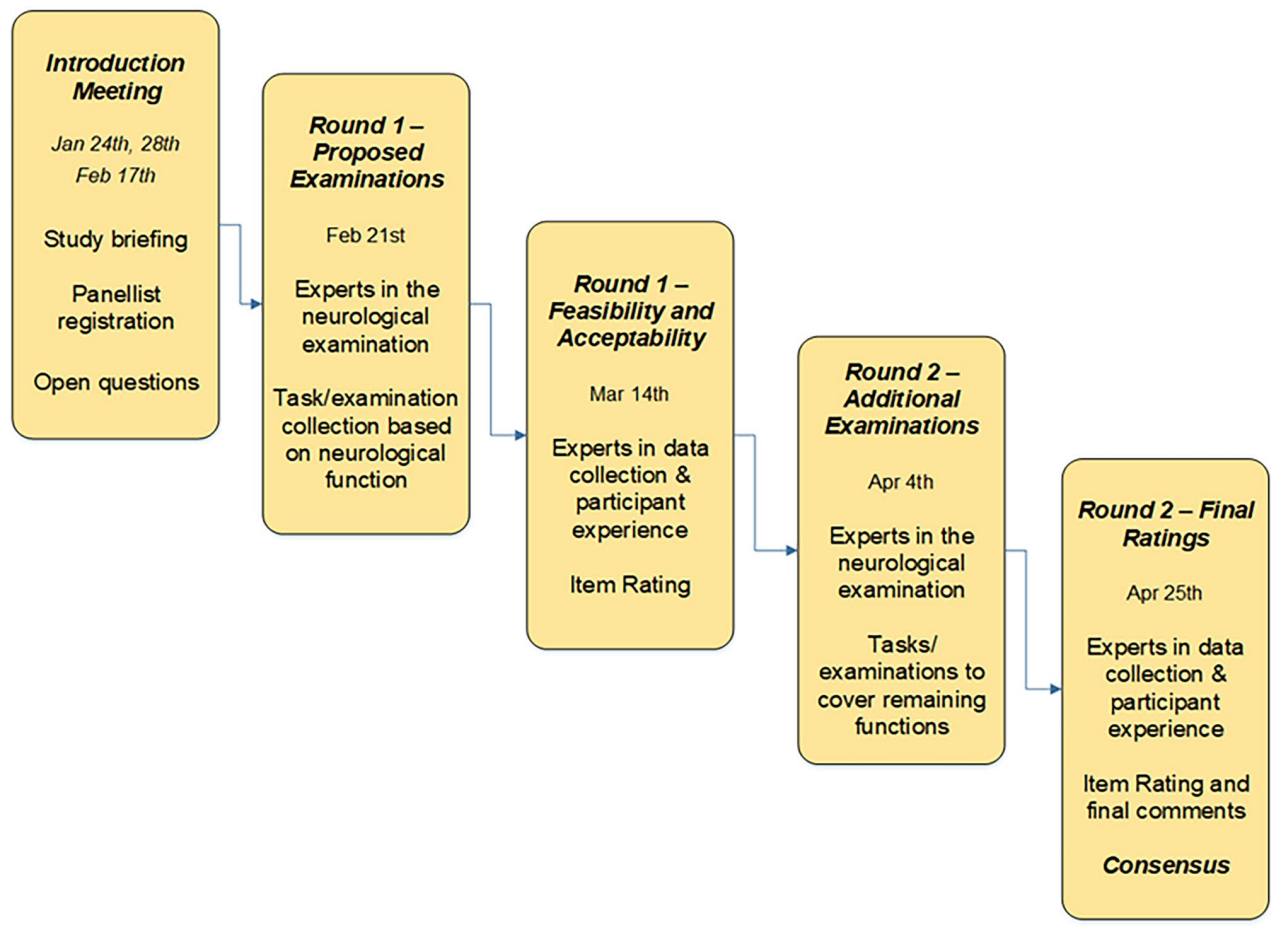
Description of the Delphi rounds and timetable

Initial online meetings with a round of introductions were conducted to explain the scope of the study and what was required by each group of experts. These session were followed by questions and answers. Shortly after, questionnaires were circulated, and panellists had 2 weeks to consider the questionnaires and return their answers, with a reminder being sent every two working days, on Tuesdays and Thursdays. The choice of this time window to return the answers was to maintain momentum and to avoid panellist withdrawal. We were able to gather a 100% response rate at the end of each round, with no withdrawals after the Delphi start. Panellists were contacted via email on blind carbon copy (BCC) throughout the rounds. A flowchart for each of the Delphi rounds is represented in Fig. 2. All data were collected and stored through the Qualtrics XM platform. Raw data for this paper will not be available for further use, but the final NeuroEpiTool will be freely available to use. A table describing the questionnaires is provided in Table 1. The entire exercise was conducted in English.

**Table 1:** Description of Delphi questionnaires

Round	Aim	Panellist	Question type	Question
1.1	Neurological examination content	Expert in neurological examination	Open ended	Identify three neurological tests that maximise your ability to rule out neurological impairment and clarify the impairment they rule out
1.2a	Neurological examination feasibility	Expert in data collection	Closed answer	How would you rate (on a scale from 0 to 4) the feasibility of the following neurological tests, in terms of complexity, to be performed and interpreted by any health worker?
1.2b	Neurological examination acceptability	Expert in participant experience	Closed answer	How would you rate (on a scale from 0 to 4) the acceptability of the following neurological tests, in terms of avoiding discomfort for participants?
2.1	Neurological examination content	Expert in neurological examination	Open ended	Provide additional tests to cover the remaining functions. In addition, what are three anamnestic questions you would ask to rule out neurological impairment?
2.2a	Neurological examination feasibility	Expert in data collection	Closed answer	How would you rate (on a scale from 0 to 4) the feasibility of the additional neurological tests and anamnestic questions, in terms of complexity, to be performed/asked and interpreted by any health worker?
2.2b	Neurological examination acceptability	Expert in participant experience	Closed answer	How would you rate (on a scale from 0 to 4) the acceptability of the additional neurological tests and anamnestic questions, in terms of avoiding discomfort for participants?

#### Round 1

The aim of the first round was to identify the tests able to rule out the largest number of neurological signs when conducted on a participant coming from the general population and to assess the feasibility and acceptability of administering them. The questionnaire was composed of open-ended questions, and experts in neurological examination provided a title and short description of a maximum of three tests accompanied by a description of the neurological impairment ruled out, if negative. Data acquired were then collected and organised (i.e. tests were listed and described; some items were merged) to be circulated to the experts in data collection and participant experience.

Experts in data collection rated the proposed tests in terms of the feasibility of them being performed and correctly interpreted by a trained health worker on a real-life participant. They rated feasibility on a Likert-type scale from 0 to 4 (e.g. *extremely easy to be performed and interpreted by any health worker/extremely difficult to be performed and interpreted by any health worker*). Similarly, the same tests were presented to the experts in participant experience, who evaluated their acceptability by the potential participant, answering in a Likert-type scale from 0 to 4 (e.g. *extremely comfortable/extremely uncomfortable*).

#### Round 2

All tests collected during Round 1 were mapped against the framework of the neurological function shown in Fig. 1. This was circulated to the experts in the neurological examination for them to identify and suggest additional tests to fill any gap. Additionally, experts were invited to provide comments on the neurological function mapping. In this round, the experts in neurological examination were also asked to identify a maximum of three anamnestic questions each. The aim of the questions was—as for the tests—to rule out the largest possible neurological impairment. The anamnestic questions were aimed at assessing symptoms which could not be assessed with a test (e.g. headache). Afterwards, the experts in data collection and participant experience once again rated each of the new tests or anamnestic questions on the basis of their feasibility in administering/interpreting and acceptability. The rounds were then closed and the Delphi completed. Experts were always encouraged to provide their own feedback on the Delphi exercise, the questionnaires provided, and the neurological function mapping.

### Achieving consensus

The Delphi leaders (VRF, VG) collated all data and delivered them back to the experts according to their role, from round to round. To reach a final consensus on each of the tests/questions, a threshold of at least an average 2.5 rating out of 4 for both feasibility and acceptability had been established. Tests/questions whose acceptability was ranked between 2 and 2.5 out of 4 were deemed to be potentially included albeit with a special warning to the assessor (e.g. “the following test may cause discomfort to the participant, please consider their willingness to perform the test”).

## Findings

After sending out invitations via email, thirteen panellists agreed to participate in the study. Before the start of the Delphi, one expert in the neurological examination withdrew from the study, leading to a final sample of twelve panellists (six experts in the neurological examination, five experts in data collection - two of them also experts in the neurological examination, and three in participant experience).

An overview of the main findings and ratings for each test and anamnestic question can be found in Table 2, and the final selection of tests and questions is described in Table 3. A mapping of the neurological functions covered by the selected tests is represented in Fig. 1. A total of fourteen different tests were provided by the experts in the neurological examination (nine in the first round and five in the second round), with a mean feasibility rating of 2.89 out of 4 (± 1.22) and mean acceptability rating of 3.00 out of 4 (± 1.10).Three tests were excluded as they did not reach the feasibility threshold, leaving eleven tests with a mean feasibility rating of 3.16 (± 1.04) and mean acceptability rating of 3.00 (± 1.08). Out of these eleven tests, two assessed a similar neurological function (nose-index and finger chase), and therefore the one with the highest ratings was prioritised, leading to a final selection of ten tests to be included. One test had an acceptability rating of 2.3 (superficial sensitivity), and therefore included accompanied by a warning.

**Table 2:** Ratings of feasibility and acceptability of the proposed items

Test title (ranked)	Feasibility rating Mean (± SD)	Acceptability rating Mean (± SD)
Neurological tests		
Nose-index	3.8 (± 0.4)	3.7 (± 0.6)
Mingazzini/pronator drift	3.6 (± 0.5)	3.0 (± 1.0)
Sitting and standing	3.6 (± 0.5)	3.3 (± 0.5)
Finger chase^a^	3.4 (± 0.9)	3.0 (± 1.0)
Walking on a line	3.2 (± 0.4)	2.7 (± 1.2)
Cognition (MMSE/MoCA)	3.0 (± 1.7)	3.0 (± 1.7)
Superficial sensitivity	3.0 (± 1.2)	2.3 (± 1.2)^b^
Deep sensitivity	2.8 (± 1.1)	3.3 (± 1.1)
Smile/strong eye closing	2.8 (± 1.6)	3.0 (± 1.0)
Visual field	2.6 (± 1.5)	2.7 (± 1.2)
Eye movement	2.6 (± 1.1)	3.0 (± 1.0)
Upper limb motor strength^c^	*2.2 (*± *1.6)*	*3.0 (*± *1.7)*
Swallowing^c^	*2.2 (*± *0.8)*	*2.7 (*± *0.8)*
Muscle tone evaluation^c^	*1.6 (*± *1.5)*	*3.3 (*± *1.5)*

Anamnestic question title (ranked)	Feasibility rating *M* (± SD)	Acceptability rating *M* (± SD)
Anamnestic questions		
Tremor noticed	4.0 (± 0.0)	2.7 (± 1.5)
Difficulty in speaking	3.4 (± 1.5)	3.3 (± 2.3)
Difficulty in swallowing	3.4 (± 0.5)	3.3 (± 1.2)
Pain & Headache	3.0 (± 1.0)	3.3 (± 1.2)
Walking disturbances	2.8 (± 1.1)	3.3 (± 1.2)
Dizzy/unsteady	2.8 (± 1.3)	3.3 (± 1.2)
Tingling	2.8 (± 1.6)	3.0 (± 1.0)
Double vision^c^	*2.4 (*± *1.3)*	*3.3 (*± *1.2)*
Numbness/loss of sensation^c^	*2.4 (*± *1.3)*	*3.3 (*± *1.2)*
Parts of body not in full control^c^	*2.4 (*± *1.3)*	*2.7 (*± *1.2)*
Pain in daily activities/sleep^c^	*2.4 (*± *1.3)*	*3.0 (*± *1.0)*
Loss of consciousness^c^	*2.2 (*± *1.6)*	*3.0 (*± *1.7)*

^a^Duplicated test/anamnestic question

^b^Rating between 2 and 2.5 (underlined)—included with warning

^c^Non-consensus items (italics)

**Table 3:** Final selection of items and their description

Item title	Description
Neurological tests	
Nose-index	Ask the participant to stretch out their arms in front of their body, palms facing upward and eyes closed Ask the participant to maintain that position for 5 s
Mingazzini/pronator drift	Upper limbs—with the participant standing, ask them to, first with eyes opened and then closed, touch the tip of their nose with both index fingers, at least 10 times (5 with each hand) Lower limbs—ask the participant to lie down on their back and try to raise both of their legs above the waist, with both knees bent. Ask the participant to stay in that position for 5 s
Sitting and standing	Ask the participant to consecutively sit and stand up from a chair, without holding on. Repeat three times in a row
Walking on a line	Ask the participant to walk in a straight line for five to ten steps, stop, then turn around and come back, first on toes and then on heels
Cognition (MMSE/MoCA)	The MMSE [29] and MoCA [30] will be adapted into the tool
Superficial sensitivity	Touch and pinch several parts (face, upper limbs, lower limbs) of both sides of the body of the participant with a cotton piece or toothpick Ask the participant if they feel it and if there is any difference from left to right
Deep sensitivity	Upper limbs—with the participants' eyes closed, touch one of their FINGERS and ask which part of their body is being touched. Move the finger up or down. Then ask if the finger is being moved up or down Lower limbs—with the participants' eyes closed, touch their BIG TOE and ask if a part of their body is being touched. If the answer is yes, further ask which one. Move the toe slightly up and down. Then ask if the toe is being moved and ask in which direction
Smile/strong eye closing	Participant closes eyes with force for a few seconds. Then, participant smiles with teeth showing
Visual field	The instructor explores the extension of the visual field of the participant, by asking them to look at the tip of the instructors' nose, and assessing the limits of the participants' visual field with the hands. Guide your hand in several points from the peripheral to central vision of the participant, and ask them if they are able to see the hand (while still looking at the tip of the nose of the instructor)
Eye movement	The participant follows the instructors' fingers with their eyes The instructor asks them to follow their finger to the left, to the right, upwards and downwards
Anamnestic questions	
Tremor noticed	In the past year, have you experienced, or has someone in your family or friends told you that they have noticed in you some kind of TREMOR?
Difficulty in speaking	In the past year, have you experienced, or has someone in your family or friends told you that they have noticed in you some kind of difficulty in SPEAKING?
Difficulty in swallowing	In the past year, have you experienced CHOKING more than usual when eating or drinking?
Pain & Headache	Pain—in the past year, have you experienced any PAIN that interfered with your daily activities or that woke you up from sleep? Headache—in the past year, have you experienced any HEADACHE that interfered with your daily activities or that woke you up from sleep?
Dizziness or Unsteadiness	In the past year, have you experienced DIZZINESS or UNSTEADINESS when standing?
Tingling	In the past year, have you experienced a TINGLING or UNUSUAL SENSATIONS (e.g. pins and needles) in any parts of your body?

A total of twelve anamnestic questions were provided by the experts in neurological examination, with a mean feasibility rating of 2.77 out of 4 (± 1.24) and mean acceptability rating of 3.10 out of 4 (± 1.20). Pain and headache were split into two independent anamnestic questions. After excluding five questions which did not pass the feasibility threshold and one question that assessed a similar function already covered by the tests, a final selection of seven anamnestic questions was included, with a mean feasibility rating of 3.06 (± 1.14) and mean acceptability of 3.10 (1.20). No anamnestic question scored low on acceptability.

## Interpretation

This Delphi exercise identified ten tests and seven anamnestic questions to rule out the largest amount of neurological impairment. These items will be used as screening steps in an eHealth tool assisting the execution of the neurological examination in the absence of a neurologist, for epidemiological research.

Overall, consensus on the tests and questions to include was easily achieved in the second round. One test (finger chase test) and one anamnestic question (walking disturbances) were eliminated for substantial overlap with another, in which the one with the highest acceptability and feasibility ratings was selected (nose-index and walking on a line, respectively). Consensus on feasibility was high, with more than half of the final selection of tests scoring higher than 3 out of 4. The main concerns on non-consensus items raised by the experts in neurological examination and the experts in data collection referred to the complexity of performing and interpreting certain tests (e.g. muscle tone) without any clinical training. Conversely, consensus on acceptability was reached with more difficulty: one test rating fell between 2 and 2.5 (deep sensitivity), prompting the decision to include the test preceded by a warning, to minimise potential distress for the participants, and therefore increasing the agency of the participant to skip it.

A general consensus was reached about using a well-validated battery test to assess the cognitive function, e.g. the Mini Mental State Examination (MMSE) [29] or the Montreal Cognitive Assessment (MoCA) [30]. This preference for a validated battery is aligned with a previous Delphi exercise to identify core items for neurology clerks, i.e. neurology students with little practice experience [31]. In this previous Delphi study, the authors also identified additional neurological tests that match the findings of the current Delphi, such as the inclusion of eye movement test, visual field, facial nerve function, and clenched teeth in the cranial nerves; the pronator drift and walking assessment in the motor function and gait; and the touch sensation in the face, upper and lower limbs [31]. However, these findings from the previous Delphi exercise were aimed at neurologists in training, not non-clinical health workers suitably involved in epidemiological research. In addition, they were not designed to be included in an eHealth tool. Other previous Delphi studies have attempted to adapt neurological assessment into eHealth, as seen in specific conditions such as spinal impairment and cerebral palsy [32, 33]. A cross-sectional study demonstrated high feasibility in performing a digitised neurological examination for multiple sclerosis patients to measure disability [34]; however, the mentioned studies either focus on clinical practice and diagnosis, or rely on highly specialised trained health workers, often neglecting their potential for research and seldom adapted as such.

The final selection of the seventeen neurological tests and anamnestic questions covered to a different extent the neurological functions depicted a priori (Fig. 1). This means that when appropriately administered to individuals coming from the general population, they should be able to rule out neurological impairment, if negative. The assessment of some neurological subfunctions, such as the reflexes, was not included due to the complexity of test administration and interpretation. In addition, the assessment of sensitivity to temperature and vibration was not included, since these two require the use of extra equipment, a prerequisite that was excluded a priori. This exclusion implies that the selective impairment of these neurological functions would be missed by the current screening tests and questions, if present. However, such a gap should not be considered a major caveat; in fact, the potential selective impairment of these functions in an otherwise neurological healthy individual would anyhow require an in-depth neurological evaluation for posing a diagnosis, an approach which is well beyond the intended epidemiological tool. On the other hand, a few of these screening tests might prompt additional assessment, if found positive. For example, failing the nose-index test might prompt an examination of strength, coordination, and deep sensitivity of the upper limbs to differentiate potential different function impairment leading to similar test failure [35, 36].

The tests and questions identified by this Delphi consensus will be used as screening items for the development of an eHealth tool able to allow the assessment of neurological impairment by non-neurologists in research settings. Where appropriate, the tool will be further developed with branched tests/questions to provide the data collector with a meaningful outcome. For example, strength impairment detected with a Mingazzini test will be further classified as mild (pronation drift), moderate (falling of one arm) or severe (inability to reach the position) [37]. The final eHealth tool will undergo firstly an inter-rater reliability testing against a clinical neurologist. Secondly, it will be tested in population-based studies against clinical records of neurological diagnoses. This will allow to select the combination of impairment that best predict a specific diagnosis (i.e. Parkinson’s disease), at the population level. However, this diagnosis will not have validity at the individual level; therefore, the tool is not intended to replace the ability of the clinical neurologist to diagnose a neurological condition based on a neurological examination.

The findings of this Delphi study support the idea that it is possible and feasible to adapt neurological screening into a research context for non-neurologists. This would lead to developing an eHealth tool starting from the results of this study to be used in epidemiological research. Furthermore, the current work highlights the importance of collecting data on signs and symptoms of neurological diseases, by emphasising that continuous epidemiological research promotes the proper development and implementation of prevention strategies to tackle disease, ultimately transforming both the clinical and research landscape.

### Strengths and limitations

The Delphi exercise included a wide variety of health workers as panellists, ranging from neurologists, to epidemiologists and nurses. The interdisciplinarity of this exercise is important considering that the aim is to devise an eHealth tool to be used in a research context, with participants from different cultures and backgrounds, and assessed by non-neurologists. In addition, by increasing heterogeneity among panellists (i.e. including different expertise backgrounds and nationalities, and people living with neurological conditions), we leveraged on different expertise and increased the likelihood of identifying tests which are feasible to be performed and interpreted by non-neurologists in everyday practice.

The overall sample size was estimated to be enough for the exercise, although a larger sample would have been preferable.

## Conclusion

This Delphi exercise reached consensus on the best screening tests/anamnestic questions to guide the development of an eHealth tool aimed at maximising the capacity of ruling out neurological impairment in epidemiological studies. These tests/questions can be performed and interpreted by health workers other than neurologists and are acceptable to participants. The current work was developed within the scope of a larger project, and in preparation for the development of the tool. Future work should focus on implementing and properly standardising the proposed selection of tests and their outcomes into an eHealth tool, to be used by researchers to capture signs and symptoms of neurological diseases. Thus, a future study will focus on the development of a tool concept following the tests and questions presented in this Delphi. The project further envisions two validation studies in which the tool will be properly tested.
